# Case Report: Whole genome sequencing of small cell ovarian carcinomas

**DOI:** 10.3389/fonc.2026.1708314

**Published:** 2026-03-31

**Authors:** Sara Daoud, Emily Tinsley, Ann Treacy, Caroline Miller, Claire Thompson, Bryan T. Hennessy, Sinead Toomey, Simon J. Furney

**Affiliations:** 1 School of Medicine, Royal College of Surgeons in Ireland (RCSI) University of Medicine and Health Sciences, Dublin, Ireland; 2 Genomic Oncology Research Group, Department of Physiology and Medical Physics, Royal College of Surgeons in Ireland (RCSI) University of Medicine and Health Sciences, Dublin, Ireland; 3 Department of Histopathology, Mater Misericordiae University Hospital, Dublin, Ireland; 4 Department of Gynaecological Oncology, Mater Misericordiae Hospital, Dublin, Ireland; 5 Medical Oncology Group, Department of Molecular Medicine, Royal College of Surgeons in Ireland (RCSI) University of Medicine and Health Sciences, Dublin, Ireland; 6 Department of Medical Oncology, Beaumont Hospital, Dublin, Ireland

**Keywords:** cancer genomics, ovarian cancer, RNA-seq, small cell carcinoma of the ovary, whole-genome sequencing

## Abstract

Small Cell Carcinoma of the Ovary (SCCO) is an extremely rare form of ovarian cancer characterised by bi-allelic mutations in the *SMARCA4* gene, a member of the SWI/SNF chromatin remodelling complex. Most previous analyses have characterised SCCO using whole exome sequencing; we present the treatment plans of two SCCO patients with post-treatment analysis of whole genome sequencing and tumour RNA sequencing which include structural variant and mutational signature analysis not previously reported in the literature for this cancer type. Both patients underwent salpingo-oophorectomy followed by BEP chemotherapy and pelvic radiotherapy leading to 34 month remission in one case though one patient died 12 months post-diagnosis. Consistent with known aetiology, we identified complete *SMARCA4* loss of function and probable *SMARCA2* expression loss in both patients. Beyond this, both tumours present remarkably low tumour mutational burdens and were microsatellite stable though one sample also showed chromosomal instability with high levels of inversions and a ploidy level of 2.8 which has not been well characterised in SCCO patients. This report contributes towards the small number of cases of SCCO that are currently documented and have their genome characterised in the literature.

## Introduction

Ovarian cancer is one of the deadliest cancers among women (1) with a fatality rate of ~65% (2). Small Cell Carcinomas of the Ovary (SCCO) account for less than 0.01% of all ovarian cancers (3) and, although rare, they are highly aggressive, lack successful treatment options due to limited clinical data and most often effect premenopausal women. SCCO comprises two histologic subtypes: a pulmonary-type tumour and a hypercalcaemic-type tumour (4). The majority of patients present with the hypercalcaemic type (SCCOHT) with the clear cell carcinoma subtype being the most common within this (5). At the mutational level, SCCOHT is characterised by inactivating *SMARCA4* (also referred to as BRG1) somatic mutations (6–8), which is a tumour suppressor within the SWI/SNF chromatin remodelling complex. Beyond *SMARCA4* mutations, SCCOHT tend to show remarkable genomic stability and low mutational burden (9). To date, genomic characterisation of SCCO has been primarily exonic (6, 7, 9) with only a very limited number of studies using whole genome sequencing. Whole genome sequencing (WGS) allows for a more comprehensive analysis of microsatellite and chromosomal instability and the potential to identify non-exonic aberrations. This paper genomically characterises two SCCO samples post treatment, one currently in remission after 3 cycles of BEP chemotherapy and pelvic radiotherapy and one deceased 12 months post diagnosis after 6 cycles of BEP chemotherapy, pelvic radiotherapy and Paclitaxel chemotherapy on recurrence, using WGS and RNA-seq data. This analysis further contributes to the low levels of SCCO samples currently available in the literature. Further understanding of the genomic contributors to SCCO will benefit the identification of at-risk individuals and the improvement of therapeutic management to reduce the high mortality rate of this disease.

## Case discussion

### Tumour presentation and treatment plan

**Figure 1:**
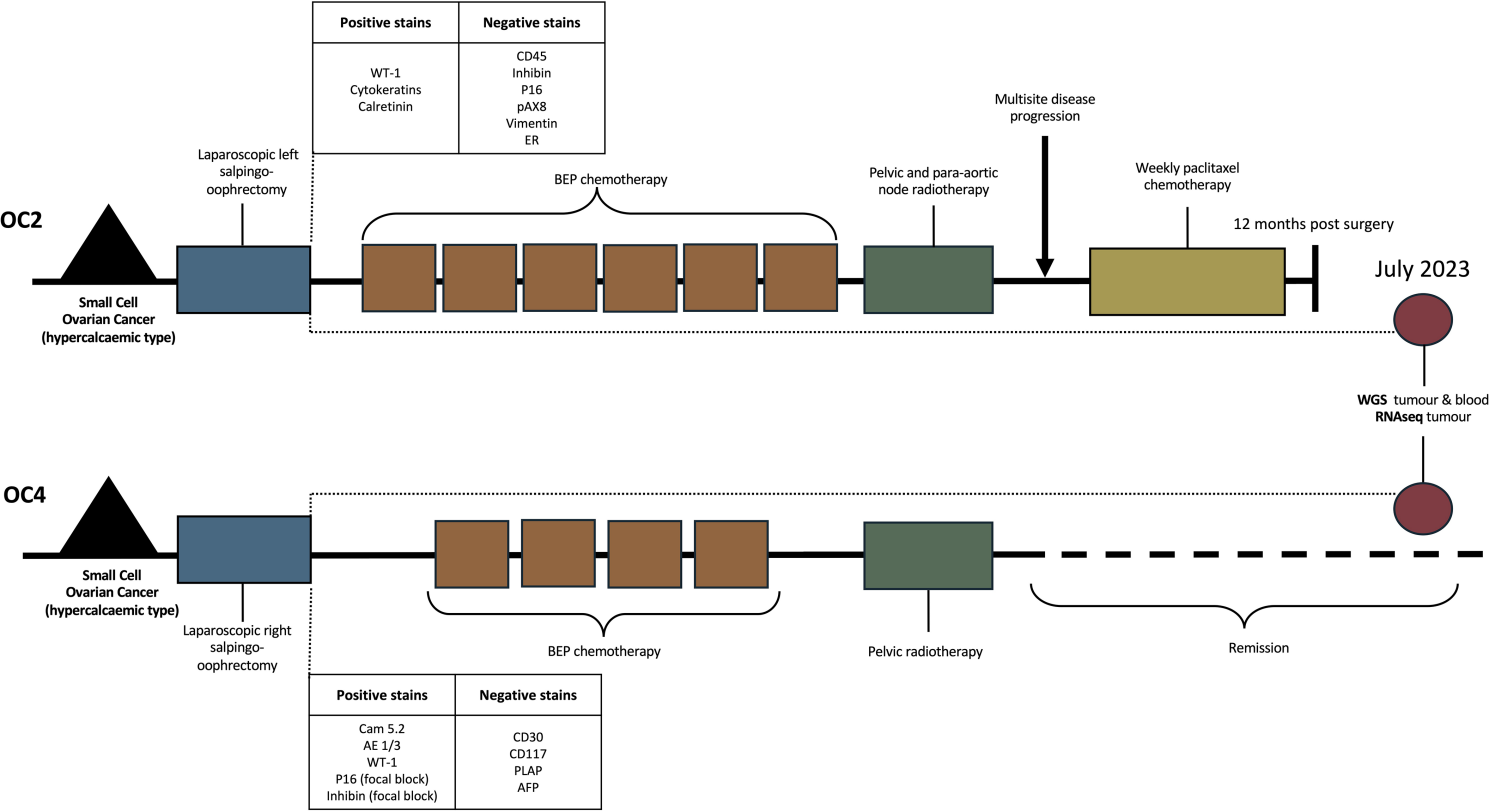
Clinical timeline and staining results from two small cell ovarian cancer patients.

Clinicians at the Mater Misericordiae University Hospital in Dublin, Ireland observed four independent SCCO cases within a span of 18 months. The diagnosis of SCCO in our series was established following comprehensive review by experienced gynaecological pathologists based on classical morphological features and clinicopathological correlation according to institutional standards at the time of care. Whilst two patients were not appropriate for further analysis, tumour samples from the remaining two patients (OC2 and OC4) were obtained and analysed. OC2 was obtained from a 24 year old female individual after a left salpingo-oophorectomy. Findings were of large volume ascites, complex 18cm haemorrhagic ovarian mass, peritoneal and omental disease. Biopsy results revealed a tumour composed of highly atypical cells occurring in sheets and trabeculae, with a focal myxoid stroma. Extensive necrosis was present and focal lymphatic space invasion as well as focal invasion of the fallopian tube were identified. Final histology confirmed as FIGO Stage IIIC disease. The patient underwent six cycles of BEP chemotherapy and external beam radiation therapy to the pelvic and para-aortic nodes however within 3 months of treatment, they had multisite disease progression and commenced Paclitaxel chemotherapy weekly (**Figure 1**). This patient deceased at 12 months following diagnosis.

OC4 was obtained from a 33 year old patient who presented with a right adrenal complex mass and similarly underwent salpingo-oophorectomy with findings of a 20cm multicystic adenexal mass. Results showed a poorly differentiated carcinoma with the tumour confined to the ovary. No extraovarian disease was identified and final histology confirmed as FIGO Stage IA. This patient underwent four cycles of BEP chemotherapy followed by pelvic radiotherapy and is currently in remission at 34 months (**Figure 1**). Histopathology analysis revealed positive staining for WT-1 and calretin in both samples with additional focal block positivity for p16 and inhibin in OC4. Proliferative index of OC4 was approximately 70%. CA125 in the blood was slightly high (62) but calcium was normal. Further staining results are summarised in **Figure 1**. Whole genome sequencing for blood and tumour of both patients was carried out in July 2023 after treatment plans were complete.

### Whole genome characterisation of small cell ovarian tumours

**Figure 2:**
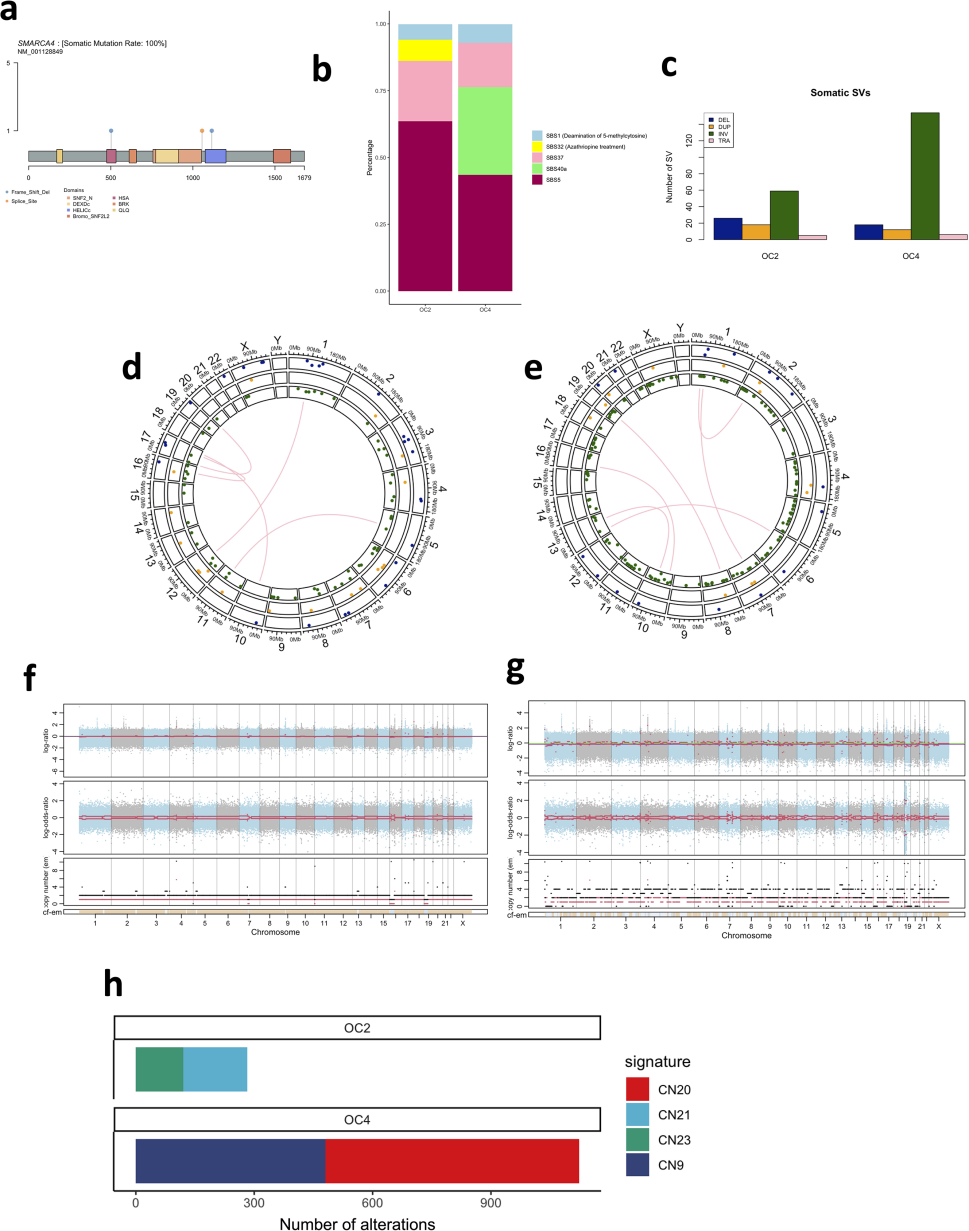
Genomic characterisation of two small cell ovarian tumours. **(a)** Lollipop plot of somatic SMARCA4 mutations. **(b)** single base COSMIC signature contributions in OC2 and OC4. **(c)** Frequency of structural variant types in OC2 and OC4.DEL, deletion; DUP, duplication; INV, inversion; TRA, transversion. **(d)** Structural variant distribution in OC2. Deletions are shown in blue, duplications in yellow, inversions in green and transversions in pink. **(e)** Structural variant distribution in OC4. **(f)** Copy number profile of OC2. **(g)** Copy number profile of OC4. **(h)** Frequency of copy number variants contributing to copy number signatures.

Somatic variant calling revealed a total of 20,163 and 12,497 small variants (small nucleotide variants and indels) across the whole genomes revealing genome-wide tumour mutation burdens (TMB) of 6.5 and 4.0 OC2 and OC4 respectively. Interestingly, only 30 (0.15%) and 34 (0.27%) of these variants occurred in exonic regions, resulting in relatively low exonic TMBs of 0.88 & 1.0 mutations/Mb for OC2 and OC4. These TMBs sit within the lower end of the average TMB observed in ovarian cancers from The Cancer Genome Atlas (TCGA) (10) and likely indicate sufficient DNA repair mechanisms which are more active within exons. Of the exonic mutations, 22 and 26 mutations were non-synonymous in OC2 and OC4 respectively. Consistent with previous analyses, we identified somatic truncating *SMARCA4* mutations in both tumours. OC2 had obtained two individual frameshift mutations in exons 9 and 24 (**Table 1**; **Figure 2a**; **Supplementary Figure 1**). Variant allele frequencies of 57.1% and 45.2% indicate homozygous loss of *SMARCA4* function via independent mutation in both alleles. Both frameshift mutations were determined to be oncogenic using the standard operating procedures for oncogenicity evaluation (10, 11) and determined to have driver status with the cancer genome interpreter (12). There were an additional 13 variants that were classified as “variants of unknown significance” including an in-frame deletion in exon 40 of the histone modifying protein *KMT2D* (p.3905_3906del) which was determined to be a driver mutation using the cancer genome interpreter (10).

**Table 1:** Summary of somatic SMARCA4 variants in two small cell ovarian cancer samples.

Sample	Position	Ref	Alt	Existing variant	HGVSp	Variant type	VAF
OC2	chr19:10994909	C	–	–	p.K502Rfs*111	Frameshift deletion	57.1%
OC2	chr19: 11027910	TA	–	–	p.Y1115Lfs*7	Frameshift deletion	45.2%
OC4	19:11025509	G	A	Rs2146498922 COSV60789716 COSV60799704	p.X1056_splice	Splice site	91.6%

Ref, reference allele; Alt, alternate allele; HGVSp, Human Genome variation Society protein level nomenclature; VAF, variant allele frequency.

OC4 obtained a single splice site variant (rs2146498922) (**Table 1**; **Figure 2a**; **Supplementary Figure 2**) which was again determined to be oncogenic and a driver mutation by the methods previously described. Additionally, SpliceAI predicted a splice donor site loss probability of 1.00 for this variant. For both patients, the absence of *SMARCA4* mutations in the blood was confirmed.

COSMIC mutational signatures showed the highest contribution for both samples coming from the clock-like signatures SBS5 (63.6% contribution in OC2, 43.5% in OC4) and SBS1 (5.8% in OC2, 7% in OC4) (13) which further indicates sufficient DNA repair mechanisms. OC2 showed a small contribution (8%) of SBS32 associated with Azathriopine treatment though the remainder of contributions came from signatures with unknown aetiology (**Figure 2b**). Double strand signatures showed major contribution of DBS17 (75% in OC2, 100% in OC4) though this signature has unknown aetiology. MSI classification determined both tumours were microsatellite stable (10). Overall, small nucleotide variant profiles of two SCCOHT samples are consistent with previous exonic analyses showing low exonic TMB, no damage of DNA damage repair deficiency and homozygous loss of function of *SMARCA4*.

Analysis of structural variants mainly revealed intra-chromosomal alterations (103 in OC2 and 184 in OC4) with a small number of translocations (**Figures 2c–e**). We identified a ~27Mb inversion on chromosome 19 in OC2 (chr19: 7445181_34401010inv) encompassing *SMARCA4* providing further potential mechanisms of *SMARCA4* interference which have not been previously characterised. OC4 showed particularly high levels of structural inversions (**Figure 2c**). In an analysis of copy number variants, OC2 showed a stable genome with a diplod ratio of -0.0308 and relative ploidy level of 2.144 (**Figure 2f**). In contrast, OC4 was much more unstable at the chromosome level (**Figure 2g**), with a diploid ratio of -0.1682 and ploidy level of 2.878. Consistent with this, copy number signatures identified enrichment of CN9 associated with diploidy and chromosomal instability (**Figure 2h**). Collectively, structural variant and copy number analysis revealed chromosomal instability and partial diploidy in one tumour sample which is not often observed in SCCOHT (9).

We used RNA-seq data to identify potential gene fusion events. This revealed a high-confidence fusion *TTC21B* and *INTS3* in OC2 and two high-confidence fusions in OC4 between *EIF1B-AS1* & *NUP54* and *NDUFA10* & *AKT2* though these were not supported by structural variant calls. Additionally previous analyses have shown that expression of *SMARCA2* is also reduced alongside mutation of *SMARCA4* (6, 9). We used RNA-seq data to calculate Transcript per million (TPM) values across genes. TPM of *SMARCA2* RNA were calculated as 4.05 and 17.80 in OC2 and OC4 respectively which is much lower than the median TPM of 44.67 reported in normal ovary samples in GTEx (14). To account for sample level differences, we ranked genes into percentiles based on TPM. Normal samples in GTEx on average have *SMARCA2* expression in the 87^th^ percentile though it was only in the 52^nd^ and 68^th^ percentile OC2 and OC4 respectively. We identified no somatic variants, copy number changes or structural variants encompassing the *SMARCA2* gene or its associated enhancers that could provide a rationale for a reduced expression often observed in SCCO.

## Discussion

In this report we classify the genomic landscapes of two SCCOHT tumours (OC2 and OC4) diagnosed and treated in Ireland between 2021 and 2023. Consistent with previous reports of SCCOHT, we identified *SMARCA4* loss of function in both samples. OC2 appeared to have lost *SMARCA4* via two novel independent frameshift mutations adding the to the current *SMARCA4* variants present in the literature. These occurred in exons 9 and 24 and as allele frequencies of ~50% were observed for both variants it indicates somatically gained compound heterozygotsity. OC4 had obtained a splice site mutation which has previously been identified in two cases of SCCOHT (7, 8). *SMARCA2* expression has been shown to contribute to SCCOHT development in conjunction with *SMARCA4* loss due to the proteins’ mutual exclusivity (6, 9). This study is limited by a lack of immunohistochemistry staining for BRG1 and BRM to confirm *SMARCA2* loss however, RNAseq was used to quantify transcripts of these genes with the caveat that this won’t always reflect true protein levels. Using RNAseq, we showed that for OC2 the expression of *SMARCA2* was indeed very low. For OC4, whilst the expression was below the median expression of normal ovary cells in GTEx, it still showed that *SMARCA2* was not fully lost which could be a result of an early stage disease. Our genomic characterization identified no aberrations that could directly explain the loss of *SMARCA2* expression indicating that it is likely a result of epigenetic changes consistent with previous reports (15). Interestingly, we did identify a somatic in-frame mutation in the epigenetic modifier *KMT2D* in one sample (OC2). *KMT2D* is a histone lysine methyltransferase primarily responsible for the activation of gene expression via mono-methylation of histones at gene enhancers (16). Whilst this variant has been labelled as having uncertain significance in ClinVar, the cancer genome interpreter did annotate it as a driver mutation. Despite this, there is no evidence of co-occurrence of *KMT2D* and *SMARCA4* mutations across any cancers in the cBioPortal database so the contribution of the variant to the development of this specific cancer is questionable. Mutations in *KMT2D* have not been previously implicated in SCCOHT but have been observed in other types of ovarian cancers (17, 18).

Beyond these mutations, exonic TMB was very low in both samples, consistent with previous SCCOHT reports (9) and mutational signatures revealed the highest contributions coming from clock-like signatures which is to be expected with *SMARCA4* driven tumorigenesis. Due to the low TMB observed in SCCO, WGS is a better predictor for mutational signature detection compared to WES, especially considering that we identified only 0.15-0.27% of variants occurring in exonic regions. Thus, WGS is beneficial in this context for confirming a lack of DNA break signatures or other pathways in this cancer type, which though eluded to, has not to our knowledge been previously shown directly.

WGS data also has the advantage of sufficient characterisation of structural variants which have not previously been looked at in analyses focusing on WES data (7, 9, 19). Contrary to previous reports of SCCOHT (20) which emphasise genomic simplicity and exclusive *SMARCA4* mutations, we did identify relatively high levels of chromosomal instability, represented mostly by inversions, and potential partial tetraploidy in OC4. Analysis of copy number signatures for this samples identified enrichment of CN9, a signature associated with chromosomal instability at the diploid level. Interestingly, CN9 has previously been associated with increased leukocyte fraction in ovarian cancer and specifically IFN-γ which induces the expression of resistant genes in cancer cells (21). We also identified enrichment of CN20 in OC4 and although the aetiology is unknown it has previously been associated with early life breast and ovarian cancer and poor prognosis (22). Additionally, chromosomal instability signatures have recently been shown to predict response to chemotherapy including platinum based treatments (23) though using different signatures to those used in this analysis (24). In the previous analysis, signatures involving specific impaired homologous recombination pathways showed the strongest association with platinum-based chemoresistance (23, 24). We present a case of SCCOHT with uncharacteristic chromosomal instability but a positive response to BEP chemotherapy leading to full remission. However, survival of this patient is also highly likely to be associated with the early stage at which it was identified as reported survival rate for FIGO stage 1 tumours is around 50% (25). OC2 progressed within 3 months of completing 6 cycles of BEP chemotherapy which was likely confounded by an advanced stage of disease burden at diagnosis and residual disease post tumour resection. This manuscript contributes towards the small number of reports currently characterising SCCOHT and describes a case of chromosomal instability in this disease which has not previously been documented. Strengths of this analysis include the availability of matched whole-genome sequencing and tumour RNA-seq data for SCCO samples which are usually sequenced only at the whole exome level. This allows for a more detailed view on genome wide changes that are not well documented for this disease. However, the analysis is limited by the small number of samples available for this disease and would benefit from matched normal RNAseq to carry out a proper differential expression analysis between normal and tumour ovaries and identify true biological changes. The accumulation of reports characterising this rare but aggressive disease will contribute towards improved understanding and optimised treatment plans.

## Patient perspective

Both patients have signed theatre consent forms which includes consenting to involvement of their samples for research and education.

## Data Availability

The datasets presented in this study can be found in online repositories. The names of the repository/repositories and accession number(s) can be found below: https://figshare.com/,account/projects/264061/articles/30112099?file=57913501.

## References

[1] Siegel RL Miller KD Jemal A . Cancer statistics, 2020. CA A Cancer J Clin. (2020) 70:7–30. doi: 10.3322/caac.21590. PMID: 31912902

[2] Sung H Ferlay J Siegel RL Laversanne M Soerjomataram I Jemal A . Global cancer statistics 2020: GLOBOCAN estimates of incidence and mortality worldwide for 36 cancers in 185 countries. CA A Cancer J Clin. (2021) 71:209–49. doi: 10.3322/caac.21660. PMID: 33538338

[3] Hu D Ma D Zhang ZJ Zhang Y Huang K Li X . Prognosis comparison between small cell carcinoma of ovary and high-grade serous ovarian cancer: A retrospective observational cohort study. Front Endocrinol (Lausanne). (2023) 14:1103429. doi: 10.3389/fendo.2023.1103429. PMID: 36742399 PMC9896785

[4] Wang J Ning Y Du Y Kang Y . Lymphadenectomy benefits small cell carcinoma of ovary: A population-based analysis. Curr Oncol. (2022) 29:7802–15. doi: 10.3390/curroncol29100617. PMID: 36290894 PMC9600050

[5] Richard Dickersin G Kline IW Scully RE . Small cell carcinoma of the ovary with hypercalcemia: A report of eleven cases. Cancer. (1982) 49:188–97. doi: 10.1002/1097-0142(19820101)49:1<188::AID-CNCR2820490137>3.0.CO;2-D 6274502

[6] Karnezis AN Wang Y Ramos P Hendricks WP Oliva E D’Angelo E . Dual loss of the SWI/SNF complex ATPases SMARCA4/BRG1 and SMARCA2/BRM is highly sensitive and specific for small cell carcinoma of the ovary, hypercalcaemic type. J Pathol. (2016) 238:389–400. doi: 10.1002/path.4633. PMID: 26356327 PMC4832362

[7] Witkowski L Carrot-Zhang J Albrecht S Fahiminiya S Hamel N Tomiak E . Germline and somatic SMARCA4 mutations characterize small cell carcinoma of the ovary, hypercalcemic type. Nat Genet. (2014) 46:438–43. doi: 10.1038/ng.2931. PMID: 24658002

[8] Ramos P Karnezis AN Craig DW Sekulic A Russell ML Hendricks WPD . Small cell carcinoma of the ovary, hypercalcemic type, displays frequent inactivating germline and somatic mutations in SMARCA4. Nat Genet. (2014) 46:427–9. doi: 10.1038/ng.2928. PMID: 24658001 PMC4332808

[9] Auguste A Blanc-Durand F Deloger M Le Formal A Bareja R Wilkes DC . Small cell carcinoma of the ovary, hypercalcemic type (SCCOHT) beyond SMARCA4 mutations: A comprehensive genomic analysis. Cells. (2020) 9:1496. doi: 10.3390/cells9061496. PMID: 32575483 PMC7349095

[10] Nakken S Fournous G Vodák D Aasheim LB Myklebost O Hovig E . Personal cancer genome reporter: Variant interpretation report for precision oncology. Bioinformatics. (2018) 34:1778–80. doi: 10.1093/bioinformatics/btx817. PMID: 29272339 PMC5946881

[11] Horak P Griffith M Danos AM Pitel BA Madhavan S Liu X . Standards for the classification of pathogenicity of somatic variants in cancer (oncogenicity): Joint recommendations of Clinical Genome Resource (ClinGen), Cancer Genomics Consortium (CGC), and Variant Interpretation for Cancer Consortium (VICC). Genet Med. (2022) 24:986–98. doi: 10.1016/j.gim.2022.01.001. PMID: 35101336 PMC9081216

[12] Tamborero D Rubio-Perez C Deu-Pons J Schroeder MP Vivancos A Rovira A . Cancer genome interpreter annotates the biological and clinical relevance of tumor alterations. Genome Med. (2018) 10:25. doi: 10.1186/s13073-018-0531-8. PMID: 29592813 PMC5875005

[13] Alexandrov LB Kim J Haradhvala NJ Huang MN Tian Ng AW Wu Y . The repertoire of mutational signatures in human cancer. Nature. (2020) 578:94–101. doi: 10.1038/s41586-020-1943-3. PMID: 32025018 PMC7054213

[14] GTEx Consortium . The genotype-tissue expression (GTEx) project. Nat Genet. (2013) 45:580–5. doi: 10.1038/ng.2653. PMID: 23715323 PMC4010069

[15] Field NR Dickson KA Nassif NT Marsh DJ . SMARCA4 and SMARCA2 co-deficiency: An uncommon molecular signature defining a subset of rare, aggressive and undifferentiated Malignancies associated with defective chromatin remodeling. Cancer Lett. (2024) 605:217282. doi: 10.1016/j.canlet.2024.217282. PMID: 39369768

[16] Froimchuk E Jang Y Ge K . Histone H3 lysine 4 methyltransferase KMT2D. Gene. (2017) 627:337–42. doi: 10.1016/j.gene.2017.06.056. PMID: 28669924 PMC5546304

[17] Hillman RT Celestino J Terranova C Beird HC Gumbs C Little L . KMT2D/MLL2 inactivation is associated with recurrence in adult-type granulosa cell tumors of the ovary. Nat Commun. (2018) 9:2496. doi: 10.1038/s41467-018-04950-x. PMID: 29950560 PMC6021426

[18] Cancer Genome Atlas Research Network . Integrated genomic analyses of ovarian carcinoma. Nature. (2011) 474:609–15. doi: 10.1038/nature10166. PMID: 21720365 PMC3163504

[19] Lin DI Chudnovsky Y Duggan B Zajchowski D Greenbowe J Ross JS . Comprehensive genomic profiling reveals inactivating SMARCA4 mutations and low tumor mutational burden in small cell carcinoma of the ovary, hypercalcemic-type. Gynecol Oncol. (2017) 147:626–33. doi: 10.1016/j.ygyno.2017.09.031. PMID: 29102090

[20] Fahiminiya S Witkowski L Nadaf J Carrot-Zhang J Goudie C Hasselblatt M . Molecular analyses reveal close similarities between small cell carcinoma of the ovary, hypercalcemic type and atypical teratoid/rhabdoid tumor. Oncotarget. (2016) 7:1732–40. doi: 10.18632/oncotarget.6459. PMID: 26646792 PMC4811493

[21] Thorsson V Gibbs DL Brown SD Wolf D Bortone DS Ou Yang TH . The immune landscape of cancer. Immunity. (2018) 48:812–830.e14. doi: 10.1016/j.immuni.2018.03.023. PMID: 29628290 PMC5982584

[22] Hadi K Yao X Behr JM Deshpande A Xanthopoulakis C Tian H . Distinct classes of complex structural variation uncovered across thousands of cancer genome graphs. Cell. (2020) 183:197–210.e32. doi: 10.1016/j.cell.2020.08.006. PMID: 33007263 PMC7912537

[23] Thompson JS Madrid L Hernando B Sauer CM Vias M Escobar-Rey M . Predicting resistance to chemotherapy using chromosomal instability signatures. Nat Genet. (2025) 57:1708–1717. doi: 10.1038/s41588-025-02233-y. PMID: 40551015 PMC12283407

[24] Drews RM Hernando B Tarabichi M Haase K Lesluyes T Smith PS . A pan-cancer compendium of chromosomal instability. Nature. (2022) 606:976–83. doi: 10.1038/s41586-022-04789-9. PMID: 35705807 PMC7613102

[25] Wens FSPL Hulsker CCC Fiocco M Zsiros J Smetsers SE de Krijger RR . Small cell carcinoma of the ovary, hypercalcemic type (SCCOHT): Patient characteristics, treatment, and outcome-a systematic review. Cancers (Basel). (2023) 15:3794. doi: 10.3390/cancers15153794. PMID: 37568608 PMC10417391

