# Supplementary material for: Case Report: Whole genome sequencing of small cell ovarian carcinomas
Source: Front Oncol. 2026 Mar 31;16:1708314. doi: 10.3389/fonc.2026.1708314 (PMC13076528; doi:10.3389/fonc.2026.1708314)
Supplement: Supplementary file 1 [file DataSheet1.pdf]

Supplementary Material

Case Report: Whole genome sequencing of small cell ovarian carcinomas

Sara Daoud[1#], Emily Tinsley[2#], Ann Treacy[3], Caroline Miller[4], Claire Thompson[4], Bryan Hennessey[5,6], Sinead Toomey[5*], Simon J Furney[2*]

Materials and methods

Sequencing, alignment and variant calling

WGS was carried out for both the tumour and blood samples along with RNA-seq of the tumour samples. Sequencing, post-processing and somatic/germline variant calling was outsourced using Novogene. Briefly, for WGS, fastq paired-end-reads were filtered to discard reads based on adapter contamination, high percentage of uncertain base readings and high proportion of low quality bases. Reads were aligned to the human reference genome (hg38) using Burrows-Wheeler Aligner v0.7.17 (1), resulting bam files sorted with Sambamba v0.7.1 and duplicate reads mark with Picard v2.18.9. Germline single nucleotide variants were identified with GATK v4.0 (2), whilst somatic variants were identified with MuTect v1.1.4 (3) and Strelka v2.9.4 (4). Copy number variants and structural varians were identified with Control-FREEC (5) and DELLY v0.8.7 (6) respectively. For RNA-seq, paired-end reads were aligned to hg38 using HISAT v2.0.5 (7) with Stringtie v1.3.3b (8) used for transcript assembly and quantification

MAF file generation and usage

VCF files were converted to MAF files using Memorial Sloane Kettering Cancer Centre's tool vcf2maf (9). Mutational profile visualisations were generated using Maftools (10) in R v4.4.1 (11).

Somatic and Germline summaries

Summaries of germline mutations were generated using the Cancer Predisposition Sequencing Reporter (12) with the following optional arguments:

--genome_assembly grch38
--panel_id 0
--ignore_noncoding

Likewise, summaries of somatic mutations including, variant annotation, TMB and microsatellite stability was generated using the Personal Cancer Genome Reporter (PCGR) (13) with the following optional arguments:

--genome_assembly grch38
--tumor_site 18
--estimate_tmb
--estimate_msi.

Whole genome tumour mutational burden calculation

Whilst exonic tumour mutational was calculated within the PCGR using all exonic mutations across a target size of 34Mb. Genome-wide TMB was calculated manually using the total number of variants across a target size of 3,100Mb.

Somatic variant signature assignment

Single-base, double-base and copy number somatic variations were characterised using COSMIC SigProfilerAssignment (14).

Genome-wide copy number visualisation

Generation of genome-wide ploidy figures were generated using FACET-suites with a sensitivity of cval=500.

Structural variant visualisation

Structural variants were filtered to those that had “PRECISE” resolution and visualised using the circlize package in R (15).

Fusion transcript analysis

For identification of fusion transcripts, fastq files were realigned using STAR v2.7.11b (16) and fusion transcript identification carried out with arriba v2.4.0 (17).

SMARCA2 expression comparison with normal samples

TPM values from RNA-seq data for 195 normal ovarian tissue samples was obtained from the GTEx database v10 (18). FPKM values for OC2 and OC4 we reconverted to TPM using the formula TPM(i) = (FPKM(i) / sum(FPKM all transcripts))*10^6. For both GTEx and cancer samples, ranks of genes were calculated and converted to percentiles using the formula: 100*(rank -1)/n.

Materials and methods referenceces

1. Li H, Durbin R. Fast and accurate long-read alignment with Burrows-Wheeler transform. Bioinformatics. 2010 Mar 1;26(5):589–95.

2. McKenna A, Hanna M, Banks E, Sivachenko A, Cibulskis K, Kernytsky A, et al. The Genome Analysis Toolkit: a MapReduce framework for analyzing next-generation DNA sequencing data. Genome Res. 2010 Sep;20(9):1297–303.

3. Benjamin D, Sato T, Cibulskis K, Getz G, Stewart C, Lichtenstein L. Calling Somatic SNVs and Indels with Mutect2 [Internet]. 2019 [cited 2024 Aug 12]. Available from: http://biorxiv.org/lookup/doi/10.1101/861054

4. Saunders CT, Wong WSW, Swamy S, Becq J, Murray LJ, Cheetham RK. Strelka: accurate somatic small-variant calling from sequenced tumor-normal sample pairs. Bioinformatics. 2012 Jul 15;28(14):1811–7.

5. Boeva V, Popova T, Bleakley K, Chiche P, Cappo J, Schleiermacher G, et al. Control-FREEC: a tool for assessing copy number and allelic content using next-generation sequencing data. Bioinformatics. 2012 Feb 1;28(3):423–5.

6. Rausch T, Zichner T, Schlattl A, Stütz AM, Benes V, Korbel JO. DELLY: structural variant discovery by integrated paired-end and split-read analysis. Bioinformatics. 2012 Sep 15;28(18):i333–9.

7. Kim D, Langmead B, Salzberg SL. HISAT: a fast spliced aligner with low memory requirements. Nat Methods. 2015 Apr;12(4):357–60.

8. Pertea M, Pertea GM, Antonescu CM, Chang TC, Mendell JT, Salzberg SL. StringTie enables improved reconstruction of a transcriptome from RNA-seq reads. Nat Biotechnol. 2015 Mar;33(3):290–5.

9. Cyriac Kandoth, JianJiong Gao, Qwangmsk, Mattioni M, Struck A, Boursin Y, et al. mskcc/vcf2maf: vcf2maf v1.6.16 [Internet]. Zenodo; 2018 [cited 2025 Sep 8]. Available from: https://zenodo.org/record/593251

10. Mayakonda A, Lin DC, Assenov Y, Plass C, Koeffler HP. Maftools: efficient and comprehensive analysis of somatic variants in cancer. Genome Res. 2018 Nov;28(11):1747–56.

11. R Core Team. R: A language and environment for statistical computing [Internet]. R Foundation for Statistical Computing; 2021. Available from: https://www.R-project.org/

12. Nakken S, Saveliev V, Hofmann O, Møller P, Myklebost O, Hovig E. Cancer Predisposition Sequencing Reporter (CPSR): A flexible variant report engine for high-throughput germline screening in cancer. Int J Cancer. 2021 Dec 1;149(11):1955–60.

13. Nakken S, Fournous G, Vodák D, Aasheim LB, Myklebost O, Hovig E. Personal Cancer Genome Reporter: variant interpretation report for precision oncology. Bioinformatics. 2018 May 15;34(10):1778–80.

14. Díaz-Gay M, Vangara R, Barnes M, Wang X, Islam SMA, Vermes I, et al. Assigning mutational signatures to individual samples and individual somatic mutations with SigProfilerAssignment. Kendziorski C, editor. Bioinformatics. 2023 Dec 1;39(12):btad756.

15. Gu Z, Gu L, Eils R, Schlesner M, Brors B. circlize Implements and enhances circular visualization in R. Bioinformatics. 2014 Oct;30(19):2811–2.

16. Dobin A, Davis CA, Schlesinger F, Drenkow J, Zaleski C, Jha S, et al. STAR: ultrafast universal RNA-seq aligner. Bioinformatics. 2013 Jan 1;29(1):15–21.

17. Uhrig S, Ellermann J, Walther T, Burkhardt P, Fröhlich M, Hutter B, et al. Accurate and efficient detection of gene fusions from RNA sequencing data. Genome Res. 2021 Mar;31(3):448–60.

18. GTEx Consortium. The Genotype-Tissue Expression (GTEx) project. Nat Genet. 2013 Jun;45(6):580–5.

Supplementary Figures

Supplementary Figure 1: IGV screenshot of frameshift deletion variants in SMARCA4 in patient OC2

Supplementary Figure 2: IGV screenshot of splice site variant in SMARCA4 in patient OC4

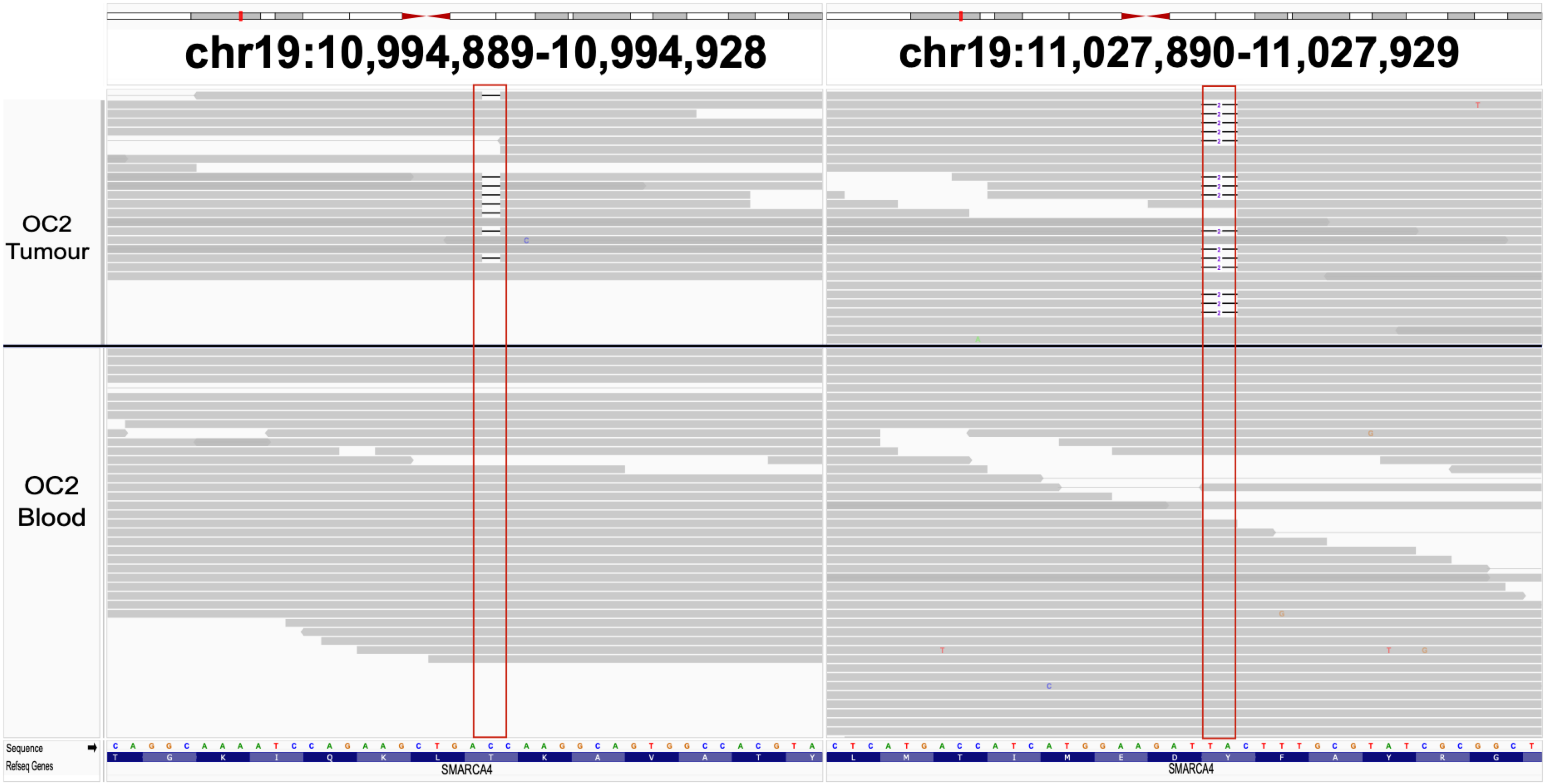
chr19:10,994,889-10,994,928
chr19:11,027,890-11,027,929
OC2 Tumour
OC2 Blood
Sequence
Refseq Genes
SMARCA4
SMARCA4

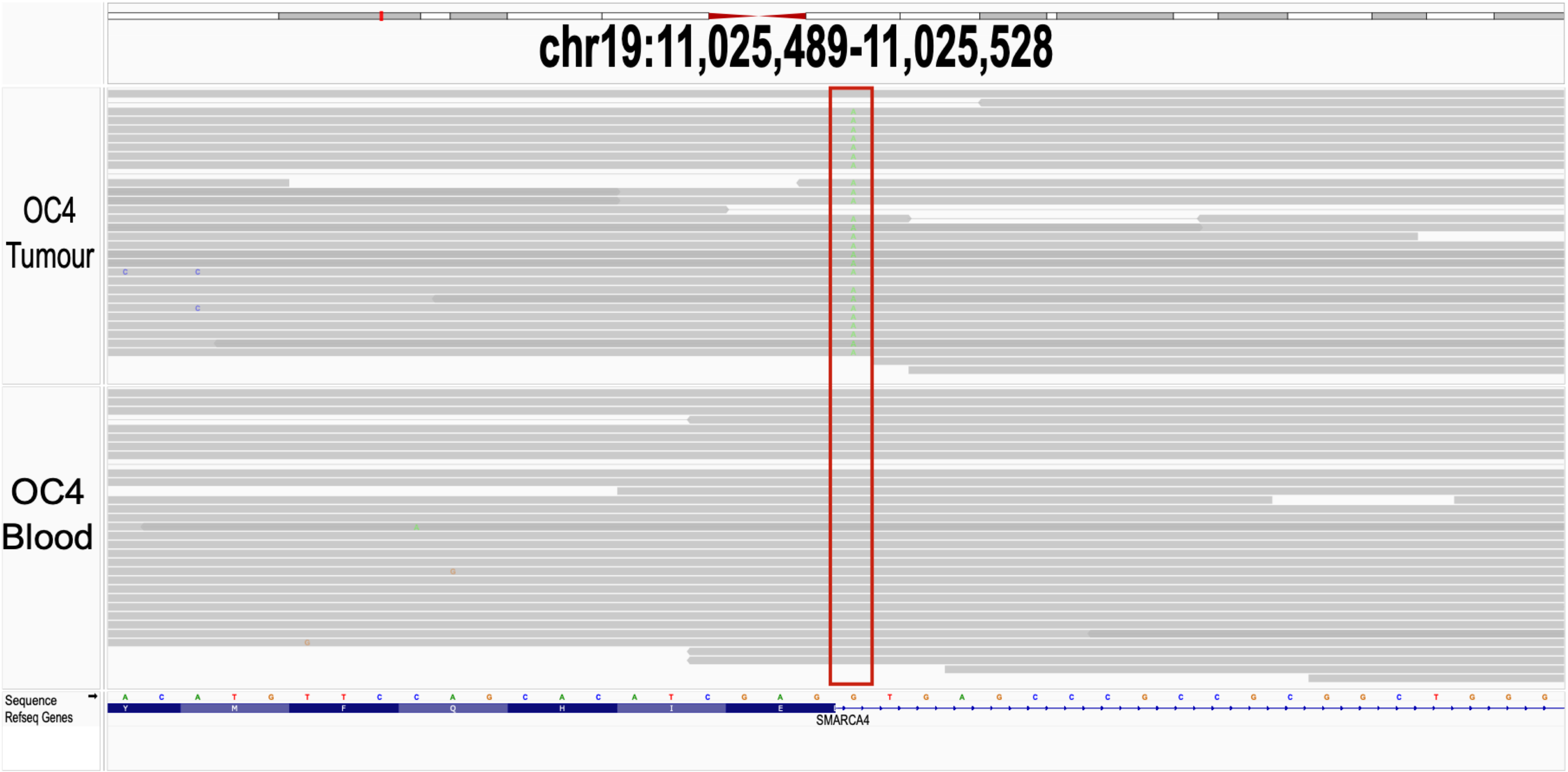
chr19:11,025,489-11,025,528
OC4 Tumour
OC4 Blood
Sequence
Refseq Genes
A C A T G T T C C A G C A C A T C G A G G T G A G C C C G C C G C G G C T G G G
Y M F Q H I E
SMARCA4
